# We are what we eat, plus some per mill: Using stable isotopes to estimate diet composition in *Gyps* vultures over space and time

**DOI:** 10.1002/ece3.8726

**Published:** 2022-03-23

**Authors:** Allan A. Baino, Grant G. J. C. Hopcraft, Corinne J. Kendall, Jason Newton, Abdelkader Behdenna, Linus K. Munishi

**Affiliations:** ^1^ Natural Resources Institute University of Greenwich Chatham Maritime UK; ^2^ Institute of Biodiversity Animal Health and Comparative Medicine University of Glasgow Glasgow UK; ^3^ 41553 North Carolina Zoo Asheboro North Carolina USA; ^4^ NERC Life Sciences Mass Spectrometry Facility Scottish Universities Environmental Research Centre East Kilbride UK; ^5^ Epigene Labs Paris 17 France; ^6^ The Nelson Mandela African Institution of Science and Technology Arusha Tanzania

**Keywords:** African white‐backed vulture, diet composition, Rüppell's vulture, stable isotopes, trophic discrimination factors

## Abstract

1. Dietary studies in birds of prey involve direct observation and examination of food remains at resting and nesting sites. Although these methods accurately identify diet in raptors, they are time‐consuming, resource‐intensive, and associated with biases from the feeding ecology of raptors like *Gyps* vultures. Our study set out to estimate diet composition in *Gyps* vultures informed by stable isotopes that provide a good representation of assimilated diet from local systems.
2. We hypothesized that differences in *Gyps* vulture diet composition is a function of sampling location and that these vultures move between Serengeti National Park and Selous Game Reserve to forage. We also theorized that grazing ungulates are the principal items in *Gyps* vulture diet.
3. Through combined linear and Bayesian modeling, diet derived from δ^13^C in *Gyps* vultures consisted of grazing herbivores across sites, with those in Serengeti National Park consuming higher proportions of grazing herbivores (>87%). δ^13^C differences in vulture feather subsets did not indicate shifts in vulture diet and combined with blood δ^13^C, vultures fed largely on grazers for ~159 days before they were sampled. Similarly, δ^15^N values indicated *Gyps* vultures fed largely on herbivores. δ^34^S ratios separated where vultures fed when the two sites were compared. δ^34^S variation in vultures across sites resulted from baseline differences in plant δ^34^S values, though it is not possible to match δ^34^S to specific locations.
4. Our findings highlight the relevance of repeated sampling that considers tissues with varying isotopic turnover and emerging Bayesian techniques for dietary studies using stable isotopes. Findings also suggested limited vulture movement between the two local systems. However, more sampling coupled with environmental data is required to fully comprehend this observation and its implications to *Gyps* vulture ecology and conservation.

## Graphical abstract

Stable isotope analyses of *Gyps* vulture tissues in East Africa revealed a large proportion of their diet is made up of grazing ungulates. δ^34^S analyses also provide baseline information for tracking vulture movement and potential applications to their conservation.

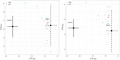

## 1 INTRODUCTION

*Gyps* vultures, African white‐backed (*Gyps africanus*), and Rüppell's (*Gyps rueppelli*) are the most abundant of the six species of vultures found in East Africa (Houston, 1990). The Rüppell's vulture is considerably larger than the African white‐backed vulture (~8.5 and ~6 kg, respectively; Houston, 1973). *Gyps* vultures are obligate scavengers that are entirely dependent on carrion resources (Mundy et al., 1992), and they feed on muscle and viscera from large animal carcasses which make up about 85% of their diet (Houston, 1990). Much of their food supply is made up of animal carcasses that have died from disease or malnutrition rather than predator kills (Houston, 1974, 1976).

Vultures contribute to nutrient recycling processes and disease regulation in our ecosystems and yet are among the most threatened taxa of birds (Ogada et al., 2012). Around 70% of vultures and other raptorial birds are categorized as threatened by the IUCN with East African *Gyps* vultures marked as critically endangered (IUCN, 2017). Declines correlate with increased incidences of poisoning, illegal trade, and loss of habitat for native herbivores which provide carrion for vultures (Ogada et al., 2012). Past telemetry and observational studies in Northern Tanzania (Serengeti‐Mara ecosystem), and more recent Ruaha‐Katavi and Selous ecosystem in Southern Tanzania, suggest distinctions in home ranges for *Gyps* and other species of vultures (Bracebridge & Kendall, 2019). North and Southern Tanzanian ecosystems, a product of habitat fragmentation, were noted from early zoological expeditions to have diverse and varied ungulate densities as distinctive features (McNaughton & Nicholas, 1986). These ungulate assemblages play an important role in maintaining vulture populations and make up a significant proportion of vulture food supply (Houston, 1974, 1976). Therefore, it is likely that there are differences in how *Gyps* vultures feed on these assemblages based on location.

In dietary analysis studies for birds of prey, estimates are based on the examination of food remains or pellets sampled at nests or resting sites (Donázar et al., 2010; Margalida et al., 2012; Real, 1996). These methods document prey items at high taxonomic resolution (Hidalgo et al., 2005; Milchev et al., 2012). However, vulture species such as those of the *Gyps* genus may ingest large amounts of meat from animal carcasses contributing less to sampled remains, and sampled remains may not be directly linked to an individual, making it difficult to establish a correlation between ingested biomass and sampled remains (Margalida et al., 2007). Subsequently, biases from this type of dietary analysis linked to sampled remains may be present in quantitative assessments of diet composition in *Gyps* vultures. Alternatively, intrinsic markers like stable isotopes can provide a good representation of assimilated diet while allowing for documented diet‐tissue isotope fractionation (Hobson & Clark, 1992). There are no published diet‐tissue fractionation estimates for δ^13^C, δ^15^N, and δ^34^S in *Gyps* vultures; however, recent developments in stable isotope ecology have enabled imputation of tissue‐specific fractionation factors through “SIDER”—a package for use in R (Healy et al., 2018).

Natural differences in stable isotope ratios in animal tissues have broad applications in ecology (Hobson, 1999). Carbon isotope ratios discriminate C3 and C4 photosynthesis in higher plants (δ^13^C = −24‰ to −34‰ and −6‰ to −19‰, respectively; Smith & Epstein, 1971), but is fairly conservative with trophic level, allowing us to estimate the contribution of C3‐ and C4‐based food sources within a consumer's tissues. δ^15^N increases with trophic level since excreted nitrogen is typically depleted in ^15^N (DeNiro & Epstein, 1981; Minagawa & Wada, 1984) allowing estimation of an animal's comparative trophic position (Gannes et al., 1998; Vanderklift & Ponsard, 2003). Sulfur isotope ratios (δ^34^S) of animal tissues are generally used to distinguish proximity to the ocean or freshwater systems since water‐derived aerosols are typically enriched in ^34^S compared with terrestrial sulfur (Newton, 2016). As with δ^13^C, δ^34^S changes little with trophic level (Δ^34^S_tissue‐diet_ = +1.2‰ for keratin; Webb et al., 2017) providing a proxy for geolocation of dietary resources.

For this study, we intended to highlight differences in how *Gyps* vultures utilize ungulate carrion and the relative contribution of ungulate carrion types to *Gyps* vulture diet as best derived by δ^13^C in Serengeti National Park and Selous Game Reserve. This study was also interested in identifying vulture movement between the two protected areas; δ^34^S a proxy for geolocation can provide an indication of feeding connectivity, as vultures have been observed in past studies to move great distances in search of food (Houston, 1974, 1976). To enrich results interpretation, we estimated tissue‐specific trophic discrimination factors (TDFs) for African white‐backed (AWB) and Rüppell's (RPV) vultures using “SIDER” and sampled blood and feathers from wild *Gyps* vultures to estimate diet composition derived from δ^13^C and movement to forage from δ^34^S. δ^13^C and δ^34^S analysis enabled us to glean and reconstruct dietary information derived from <64 days past (Kurle et al., 2013) in whole blood, to the time of the latest feather molt ~95 days (Houston, 1975), providing a time series of recent and past diets.

## 2 MATERIALS AND METHODS

### 2.1 Study area description

**FIGURE 1:**
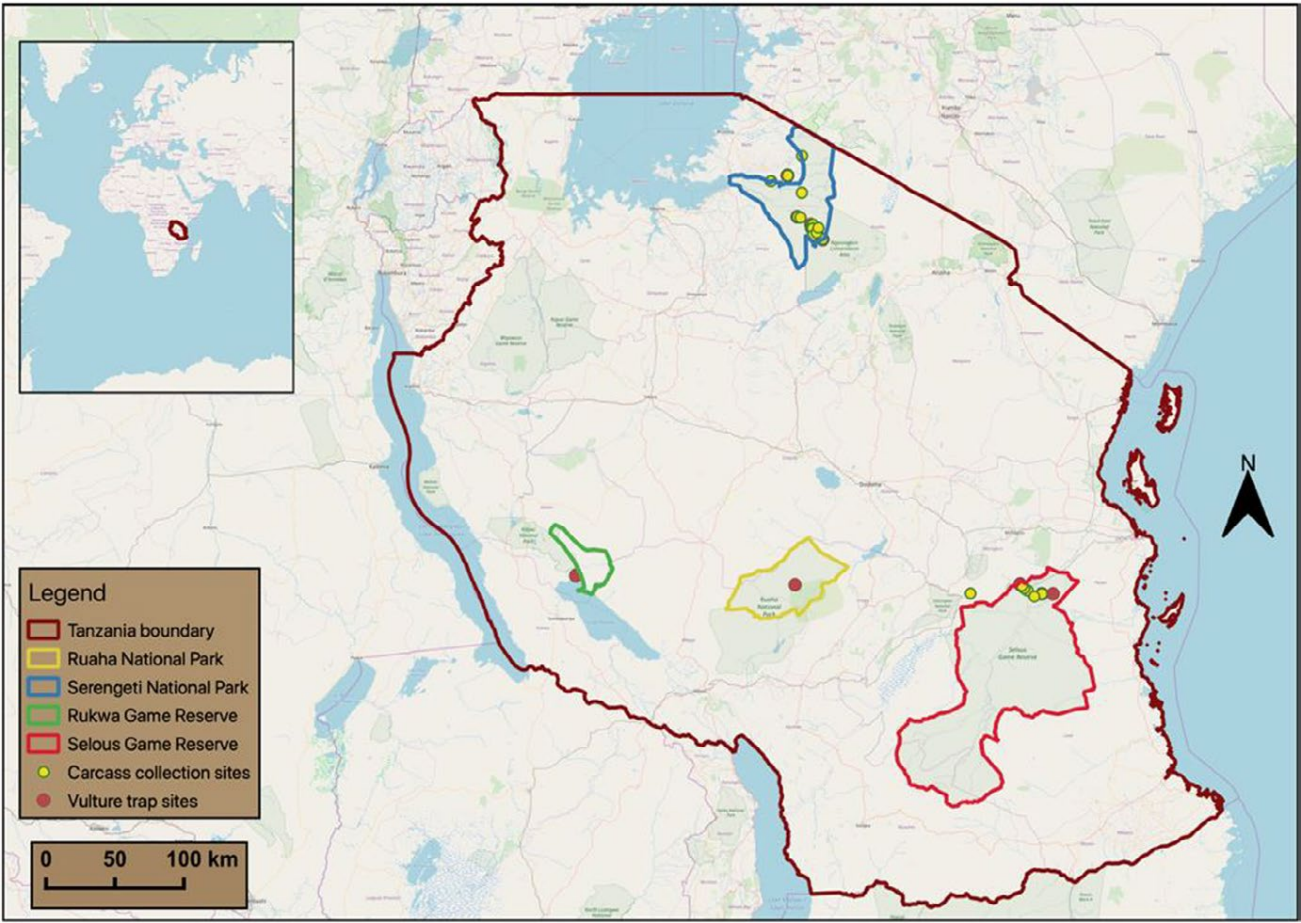
Protected areas in Tanzania where vulture and carcass tissue samples were collected

Tanzania is an East African country with some of the largest protected areas on the African continent; these areas are characterized by high diversity and densities of large mammalian (>5 kg) carnivores and herbivores, their most prominent biological feature (Keast, 1969). About 90 species of large herbivores exist on the African continent (Maglio & Cooke, 1978), with more than 20 species in large and diverse areas such as Kruger, South Africa, and Serengeti‐Mara in Tanzania and Kenya (Cumming, 1982). Pioneer studies on feeding patterns of these herbivores in Northern Tanzania noted a *graze*‐*to*‐*browse continuum* (grazers, mixed feeders, and browsers) among several species shaping animal communities (Lamprey, 1963). This observed resource partitioning played a major role in our study site selection (Figure 1), to assess how Gyps vultures utilize the *graze*‐*to*‐*browse continuum*.

Located in Northern Tanzania, Serengeti National Park (2.1540°S, 34.6857°E), experiences seasonal inundation with short and long rains from November to February and March to May, respectively (Ogutu et al., 2008). The park is a prominent grazing ecosystem (Fryxell & Sinclair, 1988) and an ecological unit defined by seasonal movement of migratory ungulates. The most numerous of these ungulates include Zebra (*Equus quagga*), Buffalo (*Syncerus caffer*), Wildebeest (*Connochaetes taurinus*), Topi (*Damaliscus lunatus*), and Thomson's gazelle (*Eudorcas thomsonii*) (Bell, 1971), which support a large proportion of vulture food supply in the Serengeti (Houston, 1974, 1976).

Selous Game Reserve (9.0000°S, 37.5000°E) in Southern Tanzania experiences a similar climate to the Serengeti. The two protected areas are a blend of savanna, shrub, and woodland vegetation, with Selous Game Reserve being predominantly a Miombo landscape (Matzke, 1971). Frequent imposition of watercourses in Selous Game Reserve's Miombo brings about interspersion of vegetation cover, which in turn creates a remarkably similar distribution of animal species and numbers (Matzke, 1971). Contrary to mass ungulate migration in Serengeti National Park, watercourses in the Selous have limited scarcity of pasture and water rendering the need for mass ungulate movements redundant (Matzke, 1971). Common ungulates in Selous are those adapted to dense woodland habitats including Elephant (*Loxodonta africana*), Buffalo (*Syncerus caffer*), Waterbuck (*Kobus ellipsiprymnus*), Black Rhino (*Diceros bicornis*), Impala (*Aepyceros melampus*), Giraffe (*Giraffa tippelskirchi*), Warthog (*Phacochoerus africanus*), and Eland (*Taurotragus oryx*) (Lamprey, 1963).

### 2.2 Data collection

Data were collected for a period of 10 months from August 2018 to May 2019, alternating between Selous Game Reserve and Serengeti National Park. We conducted vehicle reconnaissance surveys within the two protected area systems to establish suitable vulture trapping sites. We made noose lines, which are smooth fishing line (1.70 mm thick 300 lb strength) loosely tied into retractable circles along a ~1m parachute rope making a line frame (Watson & Watson, 1985). Two‐line frames were then laid loosely around provisioned or natural bait (where available) and pegged to the ground by 3‐inch × 3 mm metal pegs. Traps were set as early as 07:30 h before peak vulture food search effort which ranges between 08:00 and 12:00 h; we then retreated 50 to 60 m from trap sites to observe vulture activity.

Once vultures were noosed by their feet or neck, we rushed to the trap site, covered them with towels, and secured the birds before untying the nooses. We then proceeded to identify the species, age them by plumage, and take weight measurements. This was followed by drawing 0.5 to 1 ml of blood from tarsal veins on their feet using a 23‐gauge syringe. The blood was emptied into a labeled vacuum‐sealed, red‐topped tube, and placed in an Engel freezer (−5°C). Feather molt takes approximately 95 days per cycle and is asymmetric in primary and secondary feathers on the wings of *Gyps* vultures (Houston, 1975); therefore, we only sampled tail feathers for all individuals caught. A tail feather was cut near the dermis using straight jaw groove joint pliers. The feather was washed with still bottled water and left to dry for 3 min; it was later placed in a labeled A4 envelope. These procedures were repeated for every individual caught in Serengeti National Park: African white‐backed vulture (*n* = 12), Rüppell's vulture (*n* = 9), and in Selous Game Reserve: African white‐backed vultures (*n* = 5). Feather samples from Rukwa Game Reserve and Ruaha National Park, African white‐backed vulture (*n* = 5) and Hooded vulture (*Necrosyrtes monachus*) (*n* = 1), respectively, were provided to us by North Carolina Zoo, USA, working in those areas.

We opportunistically sampled muscle tissue from different fresh herbivore carcasses encountered along tourist circuits, anti‐poaching routes, and vulture capture sites. Muscle tissue samples in Serengeti National Park were collected from Wildebeest (*n* = 5), Eland (*n* = 1), Grant's gazelle (*n* = 2), Zebra (*n* = 9), Buffalo (*n* = 1), Hartebeest (*n* = 1), and Thomson gazelle (*n* = 1). In Selous Game Reserve, muscle samples were collected from Impala (*n* = 1), Zebra (*n* = 1), Cattle (*n* = 1), and Cape hare (*n* = 4). We cut 1 × 1 cm of muscle tissue with a surgical blade, placed it into a plastic labeled 1ml Eppendorf tube; this was then placed in our Engel freezer. Geographic coordinates for vulture trap sites and muscle tissue collection points were recorded using a GPS MAP64s (Garmin, Kansas, USA) set to datum ARC 1960.

Annual absolute counts for common ungulates were computed from transects in Serengeti National Park. A total of nine transects that cumulatively span 359.5 km in length across the park were established by the Serengeti Biodiversity Programme in 2005, to monitor long‐term wildlife population trends in the Serengeti‐Mara ecosystem. Point estimates for ungulates up to 500 m on either side of our vehicle were made along transects; age and sex composition of counts was also recorded during the surveys. Ungulate absolute counts were then grouped into three different forage categories namely Browsers, Grazers, and Mixed feeders, and ungulate counts for Serengeti National Park were gathered in tandem with vulture trapping. For Selous Game Reserve, absolute counts for 2018/2019 were acquired from the Tanzania Wildlife Research Institute (TAWIRI) census database; counts were also placed into forage categories similar to Serengeti National Park estimates. Only ungulates aged as adults during transect surveys in Serengeti National Park and from the TAWIRI database for Selous Game Reserve were compared with vulture stable isotope data for both sites.

### 2.3 Stable isotope analysis

We used a Finn pipette to remove approximately 100 μl of vulture whole blood from each of our sample vials; the blood was then emptied into 2 ml Eppendorf microtubes, frozen for 2 h, and freeze‐dried. Frozen muscle tissue samples were also freeze‐dried. Approximately 2.5 mg of vulture blood and tissue samples were weighed into 3 × 5 mm tin capsules. Vulture feather samples were cleaned with a 2:1 chloroform: methanol solution in a 100 ml beaker; they were then left to dry on white napkin tissues for 7 min at room temperature. Approximately 1 × 1 cm barb sections were cut from the pennacea proximal and basal ends of feather vanes. Feather barbs weighing ~1.3 mg were weighed into tin capsules as above.

Each sample was combusted in a PyroCube elemental analyzer (Elementar, Hanau, Germany) and then analyzed for δ^15^N, δ^13^C, and δ^34^S sequentially using an Elementar VisION IRMS at the NERC Life Sciences Mass Spectrometry Facility, East Kilbride, UK. Three internal reference materials were run every ten samples to ensure accuracy and corrected sample values for drift. These were MSAG2 (a solution of methanesulfonamide and gelatin), M2 (a solution of methionine, gelatin, glycine), and ^15^N‐labelled alanine and SAAG2 (a solution of sulfanilamide, gelatin, and ^13^C‐labelled alanine). Analytical precision (standard deviation) for international standard USGS40 was 0.03‰ and 0.08‰ for δ^13^C and δ^15^N, respectively, while for IAEA‐S1, ‐S2, and ‐S3 were 0.08‰, 1.33‰ and 0.77‰, respectively for δ^34^S. Analytical precision for internal reference materials M2, MSAG2, and SAAG2 were 0.07‰, 0.12‰, 0.04‰ for δ^13^C, 0.15‰, 0.26‰, 0.04‰ for δ^15^N and 0.90‰, 0.64‰, 0.47‰ for δ^34^S respectively. All δ^13^C, δ^15^N, and δ^34^S values reported throughout this paper follow the delta notation (McKinney et al., 1950):

$$\delta X\;\left(‰\right)=\left[\left(\frac{R_{\mathrm{sample}}}{R_{\mathrm{standard}}}\right)‐1\right]\times 1000$$

where X is ^13^C, ^15^N, or ^34^S, *R*_sample_ is the ^13^C/^12^C, ^15^N/^14^N, and ^34^S/^32^S ratios of our samples and *R*_standard_ is that of international standards V‐PDB, AIR, and CDT, respectively.

### 2.4 Statistical analyses

All analyses were carried out using R Statistical software version 4.3.0 (R Core Team, 2020) and RStudio version 1.2.1335 (RStudio Team, 2020). We calculated the absolute difference in δ^13^C, δ^15^N, and δ^34^S between pennacea proximal and basal feather barbs and plotted the data to check for individual‐level dietary differences (Figures S1–S3). We ran linear regression models to look at δ^13^C, δ^15^N, and δ^34^S variation within feather barbs by species and sampling location. We used the “ggplot2” package (Wickham, 2016) to visualize estimated categorized biomass for 23 common ungulates in Serengeti National Park from 12 months of absolute count data and Tanzania Wildlife Research Institute 2018/2019 ungulate census data. The package was also used to visualize δ^13^C, δ^15^N, and δ^34^S ratios of vulture samples across our study areas. We used the package “SIDER” (Healy et al., 2018) to fit a generalized linear phylogenetic regression model to impute AWB/RPV tissue‐specific TDF estimates. The response variables were set as δ^13^C or δ^15^N and explanatory variables and feeding ecology (carnivore) and habitat (terrestrial) set as fixed effects. The tissue type, within‐species variation (to account for numerous observations in the same species), and phylogeny were set as random effects. The models were fitted using the animal model in the package MCMCglmm with uninformative priors based on course notes within (Hadfield, 2010). MCMC chain convergence diagnostics using the Rubin–Gelman technique (Gelman & Rubin, 1992) and effective sample sizes were automatically done to assess the reliability of estimated TDFs over our four model runs. “SIDER” is unable to estimate tissue‐specific TDF for δ^34^S, and therefore, we adopted a fractionation of +1.2‰ ±0.5‰ (Webb et al., 2017).

We used δ^13^C, δ^15^N, and δ^34^S ratios in blood and feathers to parameterize general linear models (GLMs) that determined diet composition and source for *Gyps* vultures over space and time in our sampled areas. δ^13^C, δ^15^N, δ^34^S ratios as response variables varied as a function of location, vulture species, tissue type, and interaction between tissue type and vulture species. Alternate general linear models that excluded δ^13^C, δ^15^N, δ^34^S ratios in blood were run to compare the robustness of feathers in defining temporal diet variation. Data from Rukwa Game Reserve and Ruaha National Park were excluded from general linear models and all other analyses because we did not have matching blood samples for collected feather samples to make diet comparisons. We used the package “ggfortify” (Tang et al., 2016) to perform general linear model diagnostics, checking for assumptions of homoscedasticity in residuals (Figures S15–S17).

Stable isotope mixing models (SIMMs) were run with the package “MixSIAR” (Stock & Semmens, 2016) in R to determine diet contribution for vultures in Serengeti National Park. Two models were run using three bio tracers (δ^13^C, δ^15^N, and δ^34^S) with one categorical fixed variable either African white‐backed (AWB) or Rüppell's vulture (RPV). Error terms, residual error was selected for to account for potential variations in metabolic rates and/or digestibility in the different species of vultures, while process error was not selected‐for (Stock & Semmens, 2016). Prey items (herbivore muscle tissue) were combined a priori into browsers and grazers based on their feeding ecology (Phillips et al., 2005). SIMM 1 with δ^13^C, δ^15^N, and δ^34^S ratios in AWB and RPV blood, SIMM 2 with δ^13^C, δ^15^N, and δ^34^S ratios in AWB and RPV feathers were run using the “normal” MCMC parameters and model convergence was assessed using the Gelman–Rubin and Geweke diagnostics. Low muscle tissue sample sizes limited our ability to replicate comparative mixing models to estimate categorized prey item contribution to diet for vultures in Selous Game Reserve.

## 3 RESULTS

### 3.1 Regression analyses on δ^13^C, δ^15^N, δ^34^S ratios at pennacea proximal and basal feather barbs

Linear regression models indicated differences in δ^13^C, δ^15^N, δ^34^S ratios between proximal and basal feather barbs for AWBs and RPVs in Serengeti National Park (Figures S4‐S9). Only δ^34^S differences for AWB feathers sampled were significant, with proximal feather barbs having 0.6‰ higher δ^34^S than basal feather barbs (*p* = .014, see Table 1). These models also indicated AWB feather samples from Selous Game Reserve had varying δ^13^C, δ^15^N, δ^34^S between barbs (Figures S10–S12) and again only δ^34^S ratios being 0.6‰ significantly higher at proximal barbs than basal feather barbs (*p* = .009, see Table 2).

**TABLE 1:** Linear model on δ^34^S ratios in AWBs pennacea proximal and basal feather barbs sampled from Serengeti National Park

Coefficients	Estimate (‰)	*SE*	*T* value	*p*‐Value
(Intercept)	3.4463	1.8874	1.826	.0978
Proximal barbs	0.5902	0.1982	2.978	.0138

Note

*F*‐statistic: 8.866 on 1 and 10 *df*, *R* ^2^ = .47.

**TABLE 2:** Linear model on δ^34^S ratios in AWBs pennacea proximal and basal feather barbs sampled from Selous Game Reserve

Coefficients	Estimate (‰)	*SE*	*T* value	*p*‐Value
(Intercept)	4.2470	1.8505	2.295	.08339
Proximal barbs	0.6873	0.1431	4.802	.00863

Note

*F*‐statistic: 23.06 on 1 and 4 *df*, *R* ^2^ = .85.

### 3.2 *Gyps* vulture diet composition and vulture forage movement estimates from general linear models

**FIGURE 2:**
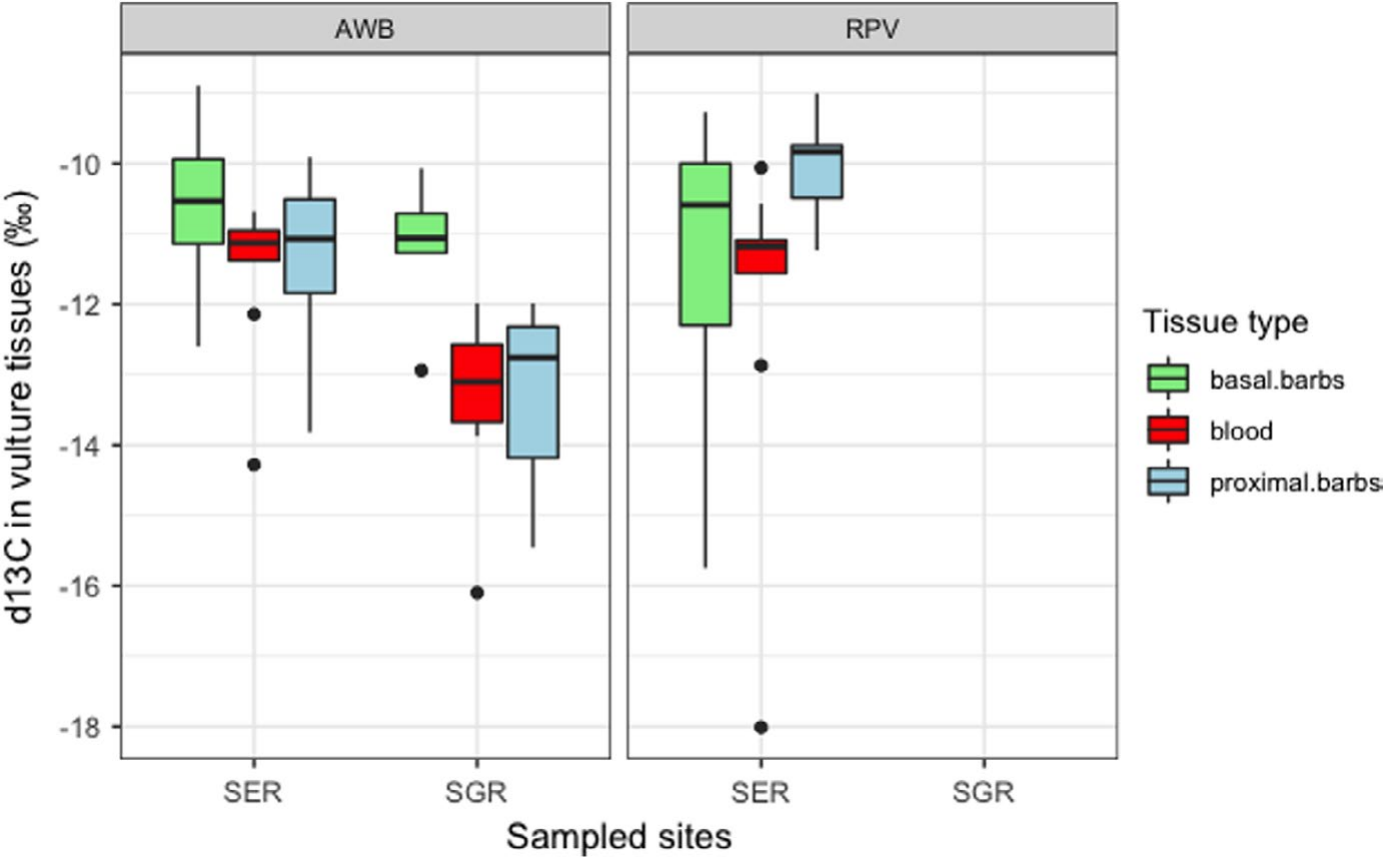
δ^13^C ratios in vulture tissues across Serengeti National Park (SER) and Selous Game Reserve (SGR) over time

**FIGURE 3:**
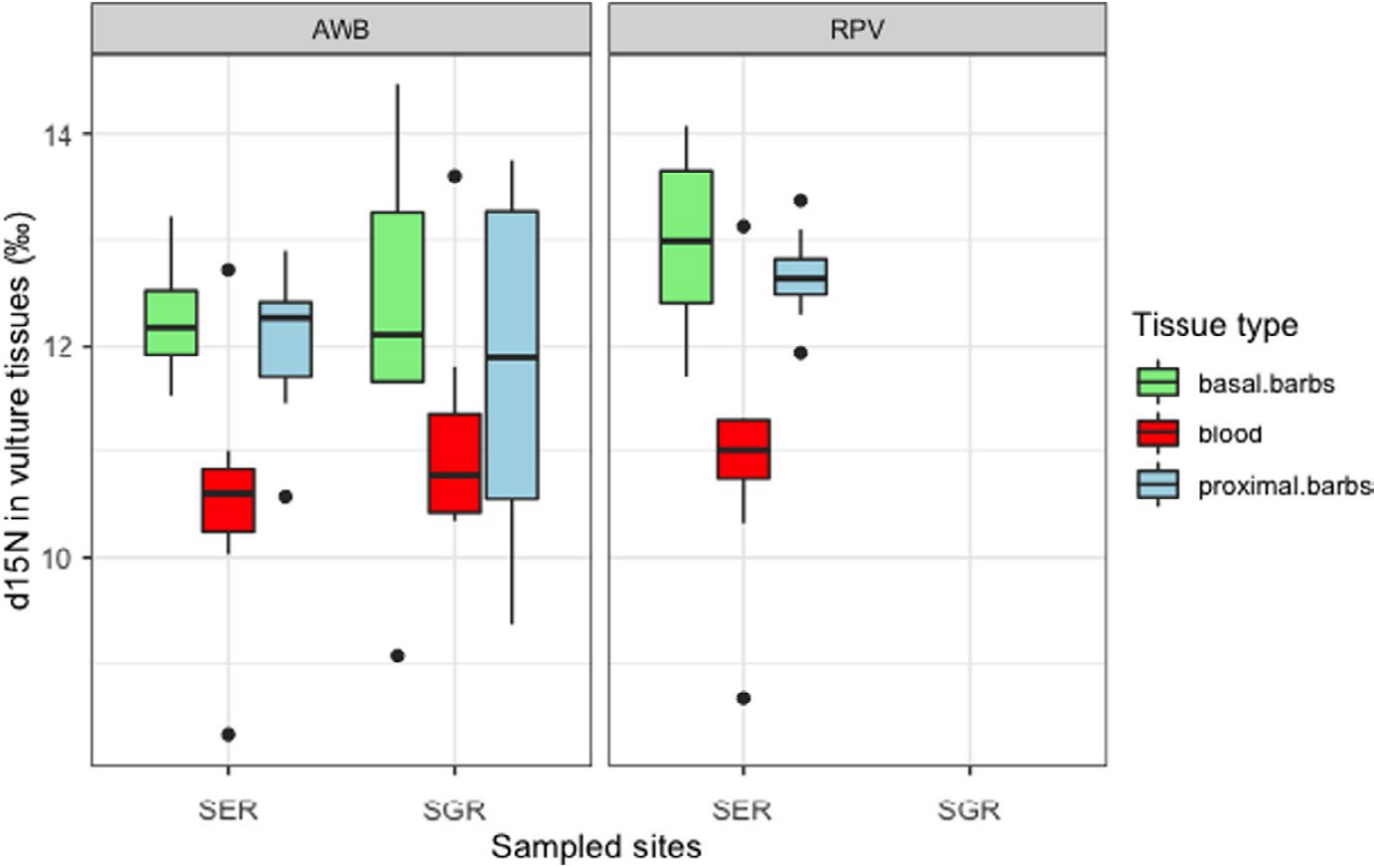
δ^15^N ratios in vulture tissues across Serengeti National Park (SER) and Selous Game Reserve (SGR) over time

**FIGURE 4:**
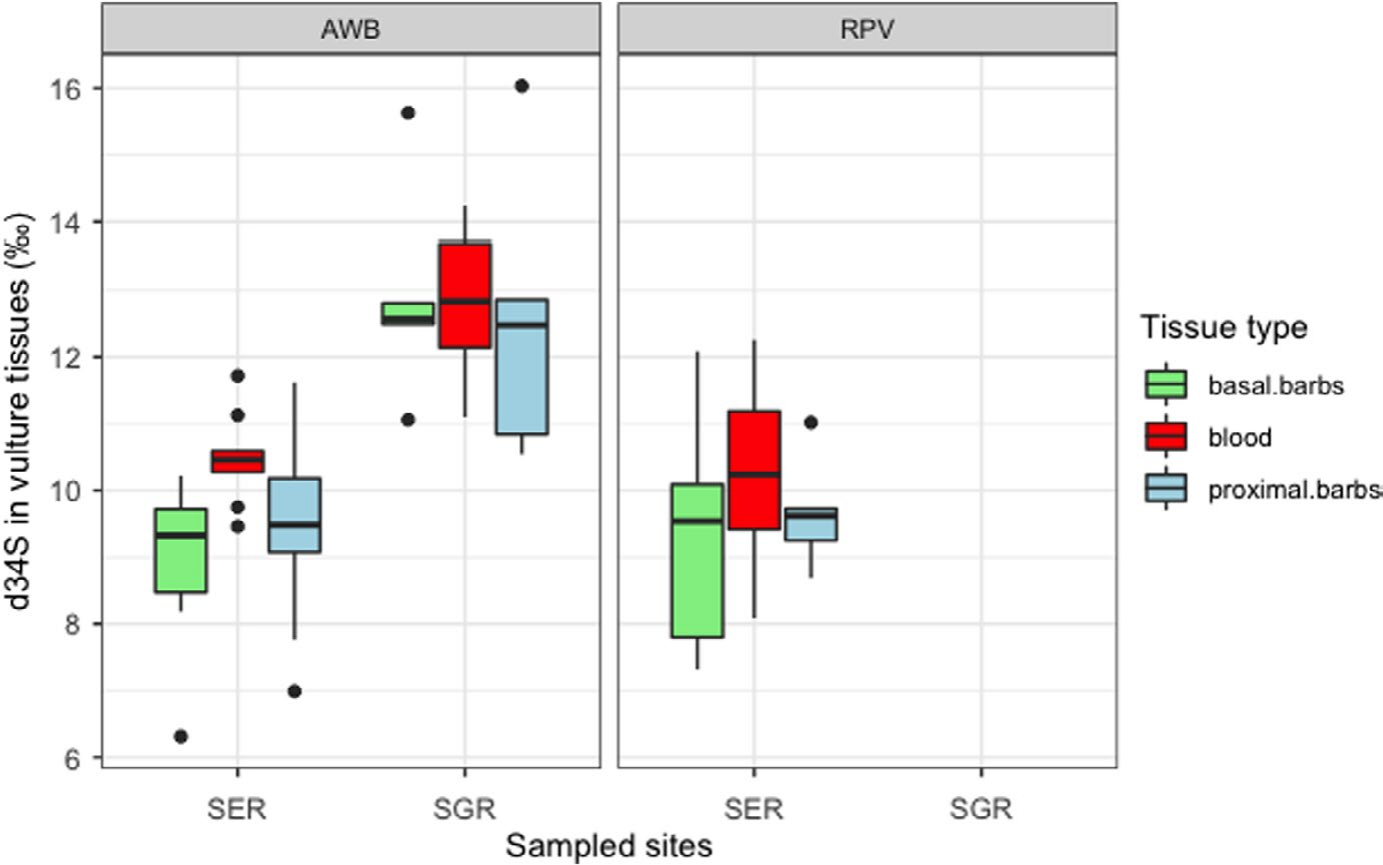
δ^34^S ratios in vulture tissues across Serengeti National Park (SER) and Selous Game Reserve (SGR) over time

General linear models informed diet composition for *Gyps* vultures derived from δ^13^C ratios consisted of grazing herbivores with variations in space and time (Figure 2). Serengeti National Park vultures fed on significantly higher proportions of grazing ungulates compared to those in Selous Game Reserve, and over time, there was a significant difference in diet given by δ^13^C ratios in blood and feathers (Table 3). The average trophic level of prey items fed on by vultures derived from δ^15^N did not vary by species and sampling location (Figure 3). However, there were significant differences over time for δ^15^N in blood, proximal, and basal feather barbs (Table 4). Vulture forage movement derived from δ^34^S ratios significantly differed across the two sites (Figure 4), and there was no indication of temporal variation in where vultures fed within their respective sampling sites (Table 5). All model residuals met the assumptions of homoscedasticity (Figures S8–S10).

**TABLE 3:** General linear model explaining diet composition derived from δ13C as a function of sampling location, vulture species, tissue type, and an interaction between vulture species and tissue type

Coefficients	Estimate (‰)	*SE*	*T* value	*p*‐Value
(Intercept)	−11.6255	0.3600	−32.297	2e^−16^
Location SGR	−1.58	0.3805	−4.152	8.42e^−05^
Species RPV	−0.3185	0.5644	−0.564	.57416
Basal barbs	1.2216	0.4622	2.643	.00995
Proximal barbs	0.2366	0.4622	0.512	.6102
Species RPV: Basal barbs	−0.6521	0.7969	−0.818	.41572
Species RPV: Proximal barbs	1.6507	0.7969	2.072	.04166

Note

Residual deviance: 154.39 on 77 DF, AIC: 305.51.

**TABLE 4:** General linear model explaining the average trophic level of prey items derived from δ^15^N as a function of sampling location, vulture species, tissue type, and an interaction between vulture species and tissue type

Coefficients	Estimate (‰)	*SE*	*T* value	*p*‐Value
(Intercept)	10.8054	0.259	41.72	2e^−16^
Location SGR	−0.051	0.2738	−0.186	.853
Species RPV	0.1187	0.4061	0.292	.771
Basal barbs	1.414	0.333	4.251	5.92e^−05^
Proximal barbs	1.068	0.333	3.212	.002
Species RPV: Basal barbs	0.59	0.57341	1.038	.30231
Species RPV: Proximal barbs	0.678	0.57341	1.184	.24014

Note

Residual deviance: 79.945 on 77 DF, AIC: 250.23.

**TABLE 5:** General linear model explaining vulture forage movement derived from δ^34^S as a function of sampling location, vulture species, tissue type, and an interaction between vulture species and tissue type

Coefficients	Estimate (‰)	*SE*	*T* value	*p*‐Value
(Intercept)	10.1	0.3148	32.022	2e^−16^
Location SGR	3.123	0.3327	9.386	2.17e^−14^
Species RPV	0.16	0.4935	0.324	.7466
Basal barbs	−0.7584	0.4042	−1.877	.0644
Proximal barbs	−0.5562	0.4042	−1.376	.1728
Species RPV: Basal barbs	−0.2724	0.6968	−0.391	.6070
Species RPV: Proximal barbs	−0.1413	0.6968	−0.203	.8399

Note

Residual deviance: 118.06 on 77 DF, AIC: 282.98.

### 3.3 Relative contribution of prey items to Serengeti National Park vulture diet

**FIGURE 5:**
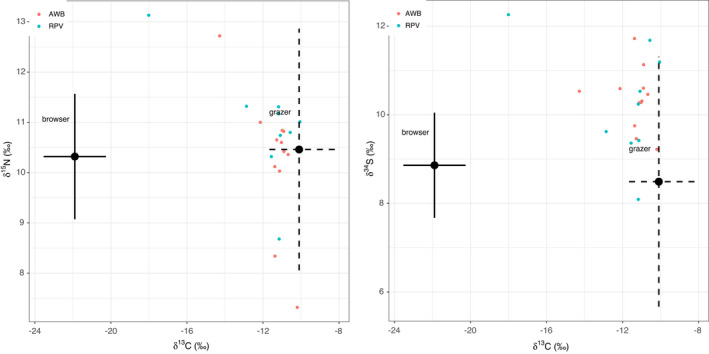
Mean isotope values (±*SD*) of δ^15^N, δ^34^S, and δ^13^C ratios in browsing and grazing herbivores showing the distribution of vulture diet derived from δ^15^N, δ^34^S, and δ^13^C ratios in AWB and RPV blood

**FIGURE 6:**
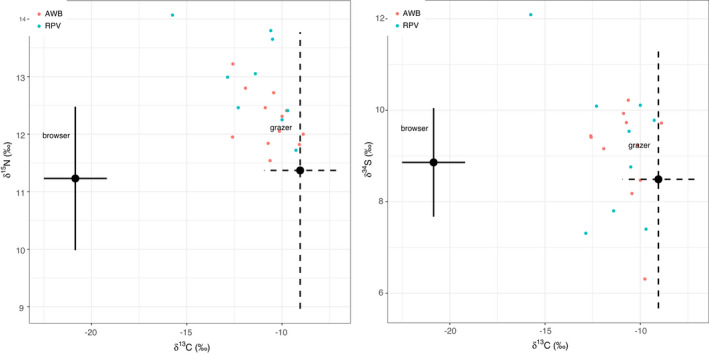
Mean isotope values (±*SD*) of δ^15^N, δ^34^S, and δ^13^C ratios in browsing and grazing herbivores showing the distribution of vulture diet derived from δ^15^N, δ^34^S, and δ^13^C in AWB and RPV feathers

Bayesian isotope mixing models estimated grazers as the principal diet item in both species of vultures sampled from Serengeti national park over time (Figures 5, 6) similar to what was observed from general linear models. Mean contribution of grazers to AWB blood diet was 90.5% ±0.05% and 9.5% ±0.05% from browsers. RPV blood diet consisted of 90% ±0.05% grazers and 10% ±0.05% browsers (Table 6). Mean contribution of grazers to AWB feather diet was 87.1% ±0.05% and 13% ±0.05% from browsers, while grazer contribution in RPV feather diet was 88.3% ±0.05% and 11.7% ±0.05% from browsers (Table 7).

**TABLE 6:** Stable isotope mixing model that used three biotracers (δ^13^C, δ^15^N, δ^34^S) in AWB and RPV blood samples from Serengeti National Park to ascertain principal diet items (SIMM 1)

	Mean	*SD*	2.5%	5%	25%	50%	75%	95%	97.5%
p.AWB.browser	0.095	0.041	0.017	0.028	0.066	0.095	0.120	0.168	0.184
p.RPV.browser	0.098	0.051	0.011	0.017	0.061	0.097	0.136	0.180	0.195
p.AWB.grazer	0.905	0.041	0.816	0.832	0.880	0.905	0.934	0.972	0.989
p.RPV.grazer	0.902	0.051	0.805	0.820	0.864	0.903	0.939	0.983	0.989

Note

DIC: 111.1316.

**TABLE 7:** Stable isotope mixing model that used three biotracers (δ^13^C, δ^15^N, δ^34^S) in AWB and RPV feather samples from Serengeti National Park to ascertain principal diet items (SIMM 2)

	Mean	*SD*	2.5%	5%	25%	50%	75%	95%	97.5%
p.AWB.browser	0.128	0.046	0.036	0.049	0.097	0.129	0.160	0.208	0.217
p.RPV.browser	0.117	0.054	0.014	0.026	0.078	0.118	0.154	0.204	0.220
p.AWB.grazer	0.872	0.046	0.783	0.797	0.840	0.871	0.903	0.951	0.964
p.RPV.grazer	0.883	0.054	0.780	0.796	0.846	0.882	0.922	0.974	0.986

Note

DIC: 95.23958.

## 4 DISCUSSION

### 4.1 Diet composition and vulture forage movement over space and time

Diet in African white‐backed and Rüppell's vultures sampled from Serengeti National Park and Selous Game Reserve consisted of C4 plant grazing herbivores. Serengeti vultures fed on grazing herbivores with higher δ^13^C values compared to those in Selous (Table 3); this was emphasized by stable isotope mixing models that estimated mean grazer contributions of at least 87% to the diet of both *Gyps* vulture species in Serengeti National Park (Tables 6 and 7). Serengeti National Park represents a surviving member of prominent grazing ecosystems in the world (Fryxell & Sinclair, 1988) whose mammalian biomass comprises 90% grazing ungulates (Bell, 1971). Furthermore, absolute ungulate counts from our transect surveys indicated more grazer abundances compared to browsing and mixed feeding ungulates, establishing the Serengeti as a grazer‐dominated ecosystem (Figure S13). It is highly likely that this grazing abundance and biomass are readily available to Serengeti *Gyps* vultures and accounted for observed elevated δ^13^C values.

Limiting resources did not permit comparative absolute categorized counts for Selous Game Reserve; however, abundance estimates for the year 2018/2019 acquired from the TAWIRI census database highlighted higher grazer counts compared to other herbivore forage categories (Figure S14). Diet composition for Selous vultures derived from δ^13^C indicated they fed on prey items that were slightly depleted in carbon compared to vultures in Serengeti (Table 3); however, that difference was within a grazing diet range (−6‰ to −19‰). There was no discernible difference between diet composition for the different species caught; we suspect this is due to similarities in the feeding ecology of *Gyps* vultures (Houston, 1990).

Temporal vulture diet comparisons for both sites derived from δ^13^C ratios in blood and feathers suggested no change in diet over time and that observed differences between blood and feather barbs from general linear models in Table 3 were representative of δ^13^C tissue‐specific fractionation. Furthermore, these differences were within predicted δ^13^C ratio offsets in *Gyps* vulture blood and feathers (Table 8) and δ^13^C fractionation estimates for the Californian Condor (New World Vulture) whole blood and feathers (Kurle et al., 2013). Therefore, we are certain *Gyps* vulture diet in the two sites consisted of grazing herbivores over 159 days (combined diet‐tissue equilibration time for blood and feathers) before the birds were sampled. The average trophic level of prey items in *Gyps* vulture diet did not vary by site and species, as was expected for African white‐backed and Rüppell's vultures that have similar feeding ecology (Houston, 1990). However, observed δ^15^N variations between vulture blood and feathers from results in Table 4 stemmed from ^15^N fractionation between sampled tissues.

**TABLE 8:** δ^13^C and δ^15^N TDFs and their associated uncertainty for AWBs and RPVs tissues from Bayesian phylogenetic regression models reported as means at 95% confidence

	δ^13^C (‰)	SD	δ^15^N (‰)	*SD*
AWB blood	0.29	1.32	2.23	1.24
AWB feather	1.38	1.34	3.21	1.27
RPV blood	0.30	1.32	2.39	1.24
RPV feather	1.23	1.34	3.17	1.26

Movement to forage as best defined by δ^34^S from vulture blood and feathers separated where *Gyps* vultures fed when comparing birds sampled in Serengeti National Park and Selous Game Reserve. *Gyps* vultures in Selous Game Reserve had higher δ^34^S values in their blood and feathers compared to vultures in Serengeti National Park (Table 5). Terrestrial sulfur ultimately results from underlying geology and the geochemical processes involved (Robinson & Bottrell, 1997); however, it can also be influenced by wind‐blown material and coastal sea spray that can be rained out (Nehlich, 2015). Biosynthetic pathways in animals bias sulfur isotope selectivity because it is locked up in large amino acids (Griffiths, 1991), causing it to fractionate less when assimilated (+1.2‰ for mammalian keratin and slightly negative for metabolically active tissues; Webb et al., 2017), making δ^34^S a moderately good proxy for geolocation. Our δ^34^S findings for vultures sampled in both sites are not consistent with published marine sulfur values (Zazzo et al., 2011), reducing the likelihood of marine resource use by vultures we sampled in both sites. Furthermore, statistical analyses on δ^34^S in whole blood and feathers did not reveal significant differences (Table 5), allowing for the assumption that our birds obtained their prey in and around their respective sampling locations over time. This, therefore, suggests our vultures did not range between sampling locations to forage at least for a period of 159 days or less before sampling, invalidating our “vultures move great distances to forage” hypothesis. However, ongoing telemetry research in Southern Tanzania has shown some tagged vultures periodically move into Northern ecosystems, shedding light on vulture movements across Tanzania (Bracebridge & Kendall, 2019). For this study, our interpretation of vulture movement was restricted to respective sampling locations, which was also limited by sample size. More isotope analyses combined with environmental data would enable definitive comprehension of the “movement to forage” theory. Matching animal tissue isotope signatures with their proximate underlying isoscapes as was done in Kabalika et al. (2020) could provide a telemetry alternative to understanding animal movement and in the case of our vultures; isotope signatures provide an opportunity to map susceptibility to risk factors by assessing their movements into areas void of protected status.

### 4.2 δ^13^C, δ^15^N, and δ^34^S variation at pennacea proximal and basal feather barbs

Regression analyses on δ^34^S in tail feathers of African white‐backed individuals by sampling location revealed significant variations, with proximal feather barbs having 0.6‰ more δ^34^S than basal barbs (Tables 1 and 2), whereas no such differences were observed in Rüppell's vultures. This 0.6‰ difference in δ^34^S ratios is within the reproducibility range of the instrument, whose analytical precision ranges between 0.47‰ and 0.90‰ using internal sulfur reference materials (see section 2.3). The exact reasons for this δ^34^S shift in African white‐backed vulture feather barbs per sampling location are less clear; however, a study in the Greater Serengeti Ecosystem predicted a sulfur isoscape with δ^34^S values ranging from +2.83‰ to +13.04‰ (Kabalika et al., 2020) consistent with δ^34^S found in our captured vultures. Therefore, sulfur differences in proximal and basal barbs for feathers of *Gyps* vultures, at least those sampled in Serengeti, is likely attributed to vulture movements to different parts of the protected area with varying δ^34^S ratios. Further analysis of δ^13^C, δ^15^N, and δ^34^S ratios for both species of vulture feather subsets revealed a significant difference in the amount of carbon at proximal feather barbs of Rüppell's vultures (1.65‰ more δ^13^C) compared to African white‐backed vultures (Table 3). The exact reasons for this species‐specific shift are beyond the scope of this work; however, the difference is likely associated with temporal shifts in diet and space use (Inger & Bearhop, 2008). The ecological significance of such differences seemingly small could for example have implications in more precise fractionation factor estimates used to ascertain relative proportions of food items in animal diet; Michalik et al. (2010) providing a better understanding of diet ecology in a species of interest.

## CONFLICT OF INTERESTS

The authors declare that they have no conflict of interest and that the views expressed herein are those of the authors.

## AUTHOR CONTRIBUTIONS

**Allan A. Baino:** Conceptualization (lead); Data curation (lead); Formal analysis (lead); Investigation (lead); Methodology (lead); Project administration (lead); Resources (lead); Software (lead); Supervision (lead); Validation (lead); Visualization (lead); Writing – original draft (lead); Writing – review & editing (lead). **Grant G. J. C. Hopcraft:** Conceptualization (lead); Formal analysis (supporting); Funding acquisition (lead); Investigation (supporting); Methodology (supporting); Project administration (lead); Resources (supporting); Supervision (lead); Validation (supporting); Writing – original draft (supporting). **Corinne J. Kendall:** Conceptualization (lead); Data curation (supporting); Funding acquisition (supporting); Methodology (equal); Supervision (supporting); Writing – original draft (supporting); Writing – review & editing (supporting). **Jason Newton:** Formal analysis (equal); Investigation (equal); Resources (equal); Software (supporting); Validation (supporting); Writing – original draft (equal); Writing – review & editing (equal). **Abdelkader Behdenna:** Formal analysis (supporting); Software (equal); Validation (equal); Visualization (equal). **Linus K. Munishi:** Conceptualization (lead); Funding acquisition (supporting); Project administration (supporting); Supervision (supporting); Writing – original draft (supporting); Writing – review & editing (supporting).

## Supporting information

Supplementary Material

## Data Availability

Data accessibility: We are what we eat, plus some per mill: Using stable isotopes to estimate diet composition in Gyps vultures over space and time: Dryad https://doi.org/10.5061/dryad.1ns1rn8qf.
